# Family-centred approaches to the prevention of mother to child transmission of HIV

**DOI:** 10.1186/1758-2652-13-S2-S2

**Published:** 2010-06-23

**Authors:** Theresa S Betancourt, Elaine J Abrams, Ryan McBain, Mary C Smith Fawzi

**Affiliations:** 1 François-Xavier Bagnoud Center for Health and Human Rights, Harvard School of Public Health, Harvard University, USA; 2 International Center for HIV/AIDS Care and Treatment Programs, Mailman School of Public Health, Columbia University, USA; 3 Department of Global Health and Social Medicine, Harvard Medical School, Harvard University, USA

## Abstract

**Background:**

Prevention of mother to child transmission (PMTCT) programmes have traditionally been narrow in scope, targeting biomedical interventions during the perinatal period, rather than considering HIV as a family disease. This limited focus restricts programmes' effectiveness, and the opportunity to broaden prevention measures has largely been overlooked.

Although prevention of vertical transmission is crucial, consideration of the family environment can enhance PMTCT. Family-centred approaches to HIV prevention and care present an important direction for preventing paediatric infections while improving overall family health. This paper reviews available literature on PMTCT programmatic models that have taken a broader or family-centred approach. We describe findings and barriers to the delivery of family-centred PMTCT and identify a number of promising new directions that may achieve more holistic services for children and families.

**Methods:**

Literature on the effectiveness of family-centred PMTCT interventions available via PubMed, EMBASE and PsycINFO were searched from 1990 to the present. Four hundred and three abstracts were generated. These were narrowed to those describing or evaluating PMTCT models that target broader aspects of the family system before, during and/or after delivery of an infant at risk of acquiring HIV infection (N = 14).

**Results:**

The most common aspects of family-centred care incorporated by PMTCT studies and programme models included counselling, testing, and provision of antiretroviral treatment for infected pregnant women and their partners. Antiretroviral therapy was also commonly extended to other infected family members. Efforts to involve fathers in family-based PMTCT counselling, infant feeding counselling, and general decision making were less common, though promising. Also promising, but rare, were PMTCT programmes that use interventions to enrich family capacity and functioning; these include risk assessments for intimate partner violence, attention to mental health issues, and the integration of early childhood development services.

**Conclusions:**

Despite barriers, numerous opportunities exist to expand PMTCT services to address the health needs of the entire family. Our review of models utilizing these approaches indicates that family-centred prevention measures can be effectively integrated within programmes. However, additional research is needed in order to more thoroughly evaluate their impact on PMTCT, as well as on broader family health outcomes.

## Background

Many programmes that aim to prevent mother to child transmission of HIV (PMTCT) in resource-limited settings have tended to take a narrow focus, often providing targeted biomedical interventions during late pregnancy and delivery, and neglecting the impact of HIV on the health of both pregnant women and families. The narrow focus of PMTCT to date represents a lost opportunity to effectively combat the vertical transmission of HIV to children - a largely preventable infection given current scientific knowledge.

We argue that family-focused approaches would facilitate broader implementation of PMTCT programming, addressing the comprehensive needs of women, particularly those in need of treatment for their own health, as well as of children and other family members, over time. This paper reviews the literature on existing models of family-centred PMTCT, as well as barriers and challenges. We lay out a vision for family-centred approaches to PMTCT and point to a number of promising new directions for preventing mother to child transmission, and also for improving overall family health and functioning and enriching the developmental context for children born into HIV-affected households.

### Family-centred care

Family-centred care has been defined in a number of ways. A useful definition comes from the American Academy of Pediatrics (AAP), which states: "In pediatrics, family-centered care is based on the understanding that the family is the child's primary source of strength and support" [1]. In this model, a number of core principles of family-centred care are outlined, including such elements as: (a) respect for each child and his or her family; (b) recognizing and building on family strengths; and (c) providing and/or ensuring formal and informal support (e.g., family-to-family support) for the child and parent(s) and/or guardian(s) during pregnancy, childbirth, infancy, childhood, adolescence, and young adulthood.

While the rubric outlined by the AAP does not perfectly translate to the context of PMTCT in resource limited settings, one might think of these principles as a more general recognition of the need to assume a family-centred approach to the treatment and maintenance of child health and wellbeing, including in the framework of PMTCT programmes. This need has been increasingly acknowledged by international organizations like the World Health Organization (WHO), which recently outlined in its *PMTCT Strategic Vision 2010-2015 *that "priority will be given to strengthening linkages between PMTCT and HIV care and treatment services for women, their children and other family members in order to support an effective continuum of care" [2].

Family-centred PMTCT models include all family members in the care paradigm and address the comprehensive health needs of all of the family members, particularly the mother and child. We propose that such an approach can facilitate the prevention of primary infection, prevention of unwanted pregnancies, ameliorate and protect the health status of the mother and child, and enrich the capacity and functioning of an HIVaffected household.

### HIV is a family illness

A family-centred approach to PMTCT has the potential to enhance health outcomes for the mother and child, as well as other members within the household. A central issue is that HIV-affected families are at high risk for a broad range of negative health outcomes, which have cascading effects on the health of all family members [3].

For example, by offering HIV testing and treatment to other family members, pregnant women may be more likely to accept HIV testing and collect their results, adhere to PMTCT regimens, and disclose their HIV-positive status to their partners [4-6]. This may result in reduced risk of vertical transmission of HIV if more women accept HIV testing, are tested earlier, and initiate treatment during an earlier time of gestation. In addition, if through appropriate support and counselling a woman shares her test results with her partner, use of condoms and other prevention methods may reduce the risk of transmission among sero-discordant couples, as well as potentially prevent transmission in future pregnancies [7,8].

In the context of PMTCT efforts to date, family-centred care has not yet fulfilled its promising potential. Coverage of HIV testing among pregnant women in low- and middle-income countries is estimated at only 21% [9]. In fact, in many high HIV burden settings, it has been difficult to identify HIV-positive women before delivery or early in their gestation, when ART or PMTCT regimens can be optimized. Access to antenatal care is routinely insufficient: only 32% of pregnant women in developing countries receive four or more antenatal care visits, the minimum number of visits recommended by the United Nations Children's Fund (UNICEF) and WHO [10].

In addition, even if a woman accesses a PMTCT regimen in a timely manner, she may not have the capacity to effectively adhere to the regimen, particularly if she is afraid to disclose her HIV status due to fear of stigma and domestic violence, or if she is lacking appropriate social support. Transportation to clinic-based follow-up antenatal care presents another significant barrier.

Similar barriers also limit access to facility-based delivery and appropriate follow up for new mothers and infants: in sub-Saharan Africa, only 40% of births take place at health care facilities; in least developed countries, this figure is as low as 32% [10]. Moreover, very few data exist on access to postnatal care, but those that are available suggest substantial shortfalls in this area, with a median coverage of 24% [11].

Mothers who have not disclosed their HIV-positive status to their partners or other family members may have difficulty pursuing an alternative to breastfeeding. Although there are promising results when offering a recent three-drug antiretroviral regimen during breastfeeding, transmission through breastfeeding cannot be completely prevented using this regimen [12]. While breastfeeding increases the risk of vertical transmission, it also avoids the social isolation that alternative safe feeding methods may evoke [13,14].

Although prevention of HIV transmission from mother to child is imperative, programmes can also improve effectiveness by addressing the overall physical and mental health of the family unit. Heymann *et al *[15] propose that infection prevention alone is insufficient in ensuring overall family health, and argue that prevention of family illness and death plays a significant role in constructing stable child-support networks. The ensuing problems of children whose parents have died from HIV disease include depression, anxiety, school drop out and high-risk sexual behaviour, placing them at greater risk of acquiring HIV infection as they transition into adulthood [15].

## Methods

### Search strategy and article selection

Using a standard review methodology, we searched PubMed, PsycINFO, and EMBASE from 1990 to November 2009 for all published articles pertaining to family-centred approaches to PMTCT. Under the global search criteria of HIV and PMTCT, returns were limited to those that contained key words or text within a matrix of relevant terminology (e.g., "family-centred"+ intervention, "MTCT-Plus"). Sensitivity of searches was improved by using key words and the bibliographies of eligible studies identified in the early stages of the search. We also asked experts in the field who have relevant subject expertise to identify additional publications or promising family-centred PMTCT programme models.

Two team members screened each of the abstracts identified, compared resultant abstracts selected, and reached consensus in order to resolve discrepancies regarding the appropriateness of inclusion for review. We included studies or programme descriptions that contained components of family-centred PMTCT in at least one of three categories of HIV care:

1. Family services provided as a part of antenatal care (ANC), such as household HIV counselling and testing, PMTCT counselling, antenatal care delivered via home visits or clinic sessions, risk assessments for intimate partner violence, evaluation for mental health problems, antiretroviral therapy (ART) for mothers or other family members as indicated, and treatment or prevention services for other illnesses

2. Family services provided around the time of birth, such as antiretroviral prophylaxis at birth for HIV-positive women, hospital-based delivery, C-section and nutritional counselling related to exclusive breastfeeding or infant formula supplementation

3. Services provided for the family following the birth of a child, such as follow up of HIV-exposed infants, family planning, early childhood development intervention activities, household risk assessment and referral or treatment for mental health problems or intimate partner violence, family HIV education or poverty-reduction strategies.

Excluded articles included those that discussed only family planning as compared to family-centred PMTCT and care, articles that were principally theoretical in nature, or articles investigating only singular biomedical interventions to prevent vertical transmission during delivery. Tables 1, 2, 3 provide an overview of models that adopt one or more family-centred components outlined in the three categories (including study design, target group, intervention components, outcomes and limitations).

**Table 1:** Family-centred PMTCT intervention models: Extension of HIV counselling and testing

Citation, country, sample size	Design	Target group	Family-centred PMTCT programme components	Outcomes	Study limitations
[4] Desgrees Du-Lou *et al*, 2009; Côte d'Ivoire; 710 women	Prospective cohort	Families	Pregnant women were encouraged to suggest HIV testing to partners Free HIV counselling and testing were provided at the request of women's partners and relatives	Prenatal HIV counselling and testing of women was followed by increased spousal communication about HIV and sexual risks, irrespective of HIV status (p < 0.01) This communication was associated with increased HIV testing in male partners (p < 0.05; OR = 4.03; 95% CI 1.50-10.82)	Study conducted among a population participating in a research programme offering routine and systematic prenatal HIV testing and counselling. Thus, the effect of counselling and testing is likely to be higher than in other community settings that do not provide systematic counselling and HIV testing
[22] Farquhar *et al*, 2004; Kenya; 2836 women and 308 men	Prospective cohort	Pregnant women and partners	Male partners were invited to voluntary counselling and testing (VCT) for HIV at an antenatal clinic Couples were offered post-test counselling Instruction was provided on contraceptive use, safe sex during pregnancy, and breastfeeding practices	Women whose partners came for VCT (10% of total) were 3 times more likely to return for nevirapine (p = 0.02), and more than 3 times more likely to report taking maternal and administering infant doses of nevirapine (p = 0.009) Couples post-test counselling was associated with an 8-fold increase in postpartum follow up and greater nevirapine utilization (p = 0.03) Couples-counselled HIV+ women were more likely to use substitute feeding methods (p = 0.03)	Women whose partners came to the clinic were a select group who may have differed from those whose partners did not come. These differences may have contributed to effects on uptake of interventions. Since 2001, the approach to PMTCT testing, and the method of drug delivery, has changed considerably
[21] Homsy *et al*, 2006; Uganda; 4462 women and 287 men	Cross- sectional	Pregnant/delivering women and partners	At a rural hospital, opt-out PMTCT education, HIV testing and counselling was provided to pregnant women in antenatal care, as well as attending partners Opt-out intrapartum HIV counselling/treatment was offered to women and partners Couples could choose to attend post-test counselling together or individually	Using this opt-out approach, HIV counselling and treatment acceptance was 97% among women and 97% among accompanying partners in the antenatal care (ANC) ward, and 86% among women and 98% among partners in the maternity ward In ANC, only 51 couples (2.8% of all tested persons in ANC) were counselled together In the maternity ward, 130 couples (37% of all tested persons in maternity) were counselled together	Staffing shortages on evenings and weekends slowed intrapartum HIV counselling and testing uptake until additional labour was hired Given the short follow-up interval, the data did not allow inference as to the rate of hospital delivery among ANC-tested HIV+ women
[20] Kakimoto *et al*, 2007; Cambodia; 20,757 women and 3714 men	Prospective cohort	Pregnant women and partners	Partners participated in a "mother class" in which information on VCT, pregnancy, delivery and newborn care was provided VCT was extended to women and their partners, and pre- and post-test couples counselling was offered	85.1% of women accompanied by partners to the mother session accepted pre-test counselling, compared with only 18.7% of women who attended the session alone (p < 0.001; OR = 25.00; 95% CI 22.7-27/8) Acceptance of post-test counselling was also higher among accompanied women (p < 0.005; OR = 1.2; 95% CI 1.07-1.37)	Pregnant women were voluntary attendees at a health facility and not randomly selected at the community level
[23] Katz *et al*, 2009; Kenya; 2104 women and 313 men total	Prospective cohort	Pregnant women and partners	Women attending an antenatal clinic were asked to invite and return with their partners to receive couples or individual VCT Males' attitudes towards VCT were evaluated, as well as the correlates of accompanying partners and receiving couples' counselling	16% of men who were informed by their wives of the availability of HIV testing accompanied their partners to the antenatal clinic Among 296 couples in which both partners received testing, 39% were counselled as a couple and 57% of men returned for a follow-up visit 87% of men attended the clinic to receive an HIV test, and 11% because they wanted information on HIV or MTCT	The study was conducted in a public antenatal clinic serving an urban population. Therefore, it may not be applicable to other resource-limited settings, including rural communities
[19] Msuya *et al*, 2008; Tanzania; 2654 women and 332 men	Prospective cohort	Pregnant women and partners	Pregnant women invited their partners to attend antenatal clinics Partners who participated in VCT received HIV, syphilis, and herpes simplex virus 2 testing, as well as pre- and post-test counselling Couples were invited to a joint counselling session	12.5% of male partners came for HIV counselling and testing 91% of HIV+ women whose partners attended VCT took nevirapine during delivery, compared with 74% of women whose partners didn't attend (OR = 3.45; 95% CI 1.00-12.00) These women were also more likely to choose not to breastfeed and adhere to a selected feeding method (OR = 3.72; 95% CI 1.19-11.63) Women's intention to disclose test results was associated with partner participation (p < 0.001; OR = 5.15; 95% CI 2.18-12.16)	Low male participation may have been due to failure of women to inform partners of VCT availability The researchers had to rely on women's self reports that they invited their partners Males may also have gone elsewhere for testing
[6] Semrau *et al*, 2005; Zambia; 9409 women and 868 men	Prospective cohort	Pregnant women and partners	Within an ongoing study on breastfeeding method and postnatal HIV transmission, women and their partners were offered couples counselling in HIV testing/PMTCT at antenatal clinics Partner involvement was promoted by community outreach	9.2% of women were accompanied by their partners for counselling Among women counselled as a couple, 96% agreed to HIV testing compared with 79% of women counselled alone (p < 0.0001). Disclosure inherent in couples counselling did not significantly increase likelihood of adverse social outcomes (e.g., intimate partner violence)	Adverse consequences of disclosure may have been underreported among women who did not disclose HIV status; thus, adverse outcomes may be overestimated by study

**Table 2:** Family-centred PMTCT intervention models: Extension of ART services

Citation, country, sample size	Design	Target group	Family-centred PMTCT programme components	Outcomes	Study limitations
[24] Byakika Tusiime *et al*, 2009; Uganda; 177 individuals	Prospective cohort	Families	At one MTCT-Plus Initiative site in Uganda, treatment and therapy for mothers and HIV-infected family members was provided, including basic treatment of HIV-related opportunistic infections, as well as provision of antiretroviral therapy (ART)	In this family-centred model, near perfect adherence to ART was observed: mean adherence in studied groups ranged from 87.7% to 100% Among adults, depression was significantly associated with incomplete adherence (p = 0.04; OR = 0.32; 95% CI 0.11-0.93)	Information was not collected on the time gap between delivery and initiation of therapeutic treatment
[25] Tonwe-Gold *et al*, 2009; Côte d'Ivoire; 605 women and 582 infants	Prospective cohort	Families	Through the MTCT-Plus Initiative, HIV prevention and care for family members, including clinical ART services Involvement and support of partners and children	Among cohort of 568 women with a living spouse, 53% disclosed HIV status to their male partner Enrolment of HIV-positive male partners was low (12%) Retention of individuals on ART was high (2.5% index women, 5.5% index partners lost to follow up)	Non-disclosure rates to partners remained high, even in the context of ART access Limited access to children outside the ANC context

**Table 3:** Family-centred PMTCT intervention models: Comprehensive Services

Citation, country, sample size	Design	Target group	Family-centred PMTCT programme components	Outcomes	Study limitations
[27] Abrams *et al*, 2007; 8 countries in sub- Saharan Africa and southeast Asia; roughly 12,000 individuals	Observational cohort	HIV-infected pregnant women and their families	As part of the MTCT-Plus Initiative, women receiving prevention of mother to child transmission (PMTCT) services were invited to enrol in MTCT- Plus, a comprehensive HIV care programme, along with their newborn infants, as well as HIV+ family and household members	More than 2/3 of index women enrolled their HIV-exposed baby or an HIV-infected family member Retention of participants was very high, with fewer than 600 adults leaving the programme, including 190 reported deaths More than 2000 infants, 90% of those who reached 18 months, were determined uninfected, and of the 761 infected children enrolled, 65% received highly active antiretroviral therapy (HAART)	The feasibility of linking the different services represented in this model may be hindered in other contexts by factors like resource constraints, human capacity and community preferences
[26] Geddes *et al*, 2008; South Africa; 2624 women	Prospective cohort	Families	PMTCT integrated into antenatal services Women were encouraged to bring partners for HIV counselling and testing Psychological services provided for discordant couples Cluster of differentiation 4 (CD4) counts measured to determine appropriate form of ART and mode of delivery Polymerase chain reaction (PCR) test given to HIV-exposed infants; HIV+ babies were enrolled in children's programme	During 18 months, 100% of women attending the clinic received counselling 91% of women and 25% of partners were tested for HIV In 338 cases of maternal HIV+, 70% of live births were by caesarean section and 98% of live babies were given nevirapine; 76% also received azidothymidine. Of the 81% of babies tested at 6 weeks (via PCR), 2.9% tested positive	May have been subject to selection bias - 11% of mothers lost to follow up Participants may have been socioeconomically and educationally better off than others who attended public facilities
[28,29] Mermin, 2005; rural Uganda; more than 6000 family members	Prospective cohort	Families	VCT for HIV extended to more than 6000 family members of HIV+ individuals Distribution of cotrimoxazole prophylaxis and a home-based water purification systems Future support for additional home-based delivery of ART for more than 4000 individuals	>95% of family members accepted VCT; 35% of married HIV+ individuals discovered they were living with an HIV- spouse Cotrimoxazole prophylaxis taken by HIV+ individuals was associated with a 46% reduction in mortality The water purification system was associated with a 25% reduction in diarrhoea among persons with HIV	Data were acquired mainly from a 2005 conference abstract and therefore have not been subjected to peer review

## Results

Our initial search of PubMed, PsycINFO, and EMBASE yielded 403 articles. Forty-eight were excluded as they were reviews. Of the remaining studies of PMTCT models, we found that 15 included at least one component of family-centred services provided before, during or after the birth of a child at risk for mother to child transmission (MTCT). Twelve were family-centred PMTCT intervention models and three were qualitative studies investigating partner perspectives on involvement in PMTCT (for qualitative studies, see Table 4[16-18]).

**Table 4:** Family-centred PMTCT intervention models: Qualitative assessments

Citation, country, sample size	Design	Target group	Family-centred PMTCT programme components	Outcomes	Study limitations
[18] Mlay *et al*, 2008; Tanzania; 18 women, 16 men, 11 counsellors	Cross- sectional	Women and men of childbearing age	Women and men were asked to identify their views concerning couples voluntary counselling and testing for HIV, couples' motivation to receive results together, and effective ways of counselling sero discordant couples	Categories identified: community sensitization; male involvement; caring; resentment; abandonment/divorce; violence Recognition of a cultural belief that ANC is exclusively for women Many participants were unaware that sero-discordancy existed	This qualitative study may have been influenced by selective enrolment and should not be viewed as a representative sample
[17] Theuring *et al*, 2009; Tanzania; 124 men	Cross- sectional	Male partners	Assessment of male attitudes regarding partner involvement in ANC/PMTCT interventions Examination of barriers preventing regular programme attendance	Among the convenience sample of males interviewed, 99% expressed positive regard for joint counselling Among males who were having children, only 46% had attended ANC/PMTCT services The primary external barrier to ANC/PMTCT services identified was "lack of knowledge and information"	Study sample of men included some individuals aged 50+ years, who are less likely to be involved in family planning
[16] Tijou Traoré *et al*, 2009; Côte d'Ivoire; 26 women and 10 men	Prospective cohort	Pregnant women and partners	Assessment of couples' decision- making process concerning infant feeding in the framework of a MTCT-Plus programme	Interviews showed that initial individual preferences were subject to conjugal negotiation, and conflicts were often resolved after revelation of HIV status to spouse Most women associated refraining from breastfeeding with an internal moral suffering; this feeling was reinforced by social pressures	Small scale of study is illustrative and not generally applicable Selective enrolment of participants who were receptive to study Attitudes may have been influenced by the project's biomedical model

Of the 12 intervention models outlined in Tables 1, 2, 3, seven focused primarily on extending HIV counselling and testing to the partners of pregnant women attending ANC clinics (see Table 1[4,6,19-23]). In some instances, uptake among partners was achieved by community outreach efforts [6], while in other contexts, women were simply encouraged to invite their partners [19]; in one hospital etting, an opt-out approach was assumed, meaning that testing and counselling were provided to partners unless they otherwise requested not to receive these services [21].

Across these studies, partner participation was associated with positive outcomes, such as greater use of antiretrovirals [19,22] and higher acceptance of post-test counselling among pregnant women [20], as well as increased spousal communication about HIV and sexual risk [4]. Moreover, when couples received pre- or posttest counselling together, greater use of alternative feeding methods [22] and greater acceptance of HIV testing [6] were observed among women. Partner participation was also often utilized as an entry point for the provision of additional PMTCT services to both male and female participants.

A second series of the studies we reviewed focused on expanding provision of antiretroviral therapy (ART) to partners and other family members (Table 2, [24,25]). One central finding in this category was high adherence and retention of ART among all participants - women, men and children - likely because of greater supports within the family unit.

A third category of studies and programme models reviewed delineated the successes and shortcomings of comprehensive PMTCT models involving numerous family members (Table 3, [26-29]). The MTCT-Plus Initiative was one of the few programmes actively seeking enrolment of family members into the programme. Overall, more than 67% of women enrolled a family member [27], primarily HIV-exposed infants born to the pregnant or postpartum woman; enrolment of other family members proved to be more challenging. At the sites in Abidjan, Cote d'Ivoire, it proved difficult to test older children within the family as they often lived with other families in rural communities. Table 3 contains detailed information pertaining to all study designs, outcomes and limitations.

Among the programme models identified in the literature review, two salient examples were the MTCT-Plus Initiative [27] and the CDC-Uganda, Global AIDS Program [28,29]. Although the conceptual framework of the MTCT-Plus Initiative is described more thoroughly in the Discussion, a number of specific results achieved by this initiative are worth noting here.

For example, among pregnant women who also enrolled their infants into MTCT-Plus Initiative programmes within the first months of life, the majority received complex antiretroviral regimens: 47% received short-course regimens during pregnancy, 20% initiated highly active antiretroviral therapy (HAART), and 30% received single-dose nevirapine. Women initiating HAART during pregnancy also exhibited an excellent immunologic response with an average increase of 451 cells/mm^3 ^after 30 months on treatment. Overall retention in care for MTCT-Plus participants initiating ART was high: 82% for pregnant women, 86% for men, and 87% of non-pregnant women at 30 months of follow up [30]. In addition, the mortality rate for both adults and pregnant women was found to be much lower than that reported at publicly funded programmes [31,32].

We were also able to acquire more recent supplementary data, which further illuminate the potential of this model to improve overall family health: from January 2003 until April 2008, 16,457 individuals (9718 adults and 6739 children) enrolled in MTCT-Plus Initiative programmes in Cameroon, Cote d'Ivoire, Kenya, Mozambique, Rwanda, South Africa, Uganda, Zambia and Thailand. Overall, 4275 (45%) women enrolled during pregnancy and 3611 (37%) women enrolled during the postpartum period.

Additionally, 1569 male partners and 449 older children living with HIV infection were enrolled and able to access comprehensive HIV care and treatment services. More than 6000 women chose to enrol their newborn child into the follow-up programme, where a battery of services, including early infant diagnosis, opportunistic infection prophylaxis, growth monitoring and antiretroviral treatment, were provided. HIV infection status was determined in more than 70% of the exposed infants, an unusually high percentage compared with traditional PMTCT programmes; approximately 10% of exposed infants were found to be HIV infected.

A second exemplary model identified through the literature review process (namely, the CDC-Uganda, Global AIDS Program [28,29]) also demonstrated a number of impressive outcomes. This programme extended home-based voluntary counselling and testing (VCT) for HIV to 6000 family members of HIV-positive individuals. The acceptance rate exceeded 95%, and 35% of those who were HIV-positive and married discovered that their spouse was HIV negative. In addition, 10% of the children under the age of five years had undiagnosed HIV.

Cotrimoxazole consumption by HIV-positive individuals was associated with a 46% reduction in mortality, and 30% to 70% lower incidence of malaria, diarrhoea and hospitalization. There was also a 63% reduction of mortality among HIV-negative children whose HIV-positive parents were taking cotrimoxazole; this finding was likely the result of reduced morbidity and mortality among the HIV-positive parents, since death of a parent was associated with a three-fold increase in risk of child mortality. Additional information the CDC programme is provided in the discussion.

## Discussion

### Barriers to effective family-centred PMTCT

In our review of published programme models/evaluations and synthesis of the available literature, a number of barriers have been identified in the implementation of PMTCT in low-resource settings, which have implications for developing effective family-centred PMTCT. These include: limited access to antenatal care and obstetric services [10,27,33]; lack of routine (opt-out) and rapid HIV testing [34-37]; poor access to CD4 monitoring [38,39]; limited access to ART, as well as to multi-drug prophylactic regimens for PMTCT [40-43]; limited testing of partners [19]; low access to paediatric testing and treatment for HIV [44,45]; and poor adherence, as well as retention in care after delivery [46].

Also problematic in the delivery of PMTCT is the lack of coordination and integration among services, such as HIV testing, counselling, and distribution of ARVs, as well as assimilation with maternal and child health services more generally [47]. In many low- and middle income countries, services are too centralized to reach remote areas, presenting a key barrier to antenatal care and PMTCT services [48,49]. In addition, as in all of health care in these countries, there is a lack of human and material resources that impacts access to care [50].

As demonstrated in our review of programme models that targeted partners for HIV counselling and testing, social support from family members must also be considered in family-centred PMTCT approaches. Women with lower levels of family support have been found to be more likely to refuse HIV testing than their peers with higher family support [51]. In addition, fear of HIV-related stigma and fears about disclosure may lead others to avoid being tested [52]. Furthermore, gender inequalities manifested in the limited education and literacy of women, power dynamics in the household about decision making, and infant care and reproductive health decisions all present barriers to family planning services and pose a significant obstacle to the primary prevention of HIV transmission in women.

For these reasons, father involvement in PMTCT and family-based testing and care are critically important. For example, as noted earlier, women who acquire HIV may be at risk for violence or abuse if they disclose their HIV-positive status to their partners without appropriate supports and engagement of their partners. These dynamics can in turn impact adherence to treatment regimens, as well as the ability of mothers to implement safe infant feeding practices [53].

### Addressing barriers by promoting a family-centred approach

#### 1. The MTCT-Plus Initiative

Of the models of family-centred PMTCT reviewed for this paper, as mentioned previously, the MTCT-Plus Initiative [27], which operated at 13 sites in eight countries in sub-Saharan Africa and in Thailand, offered one of the most comprehensive models for family-focused care using PMTCT as an entry point (see Table 3, Abrams *et al*, ref [27]). This model uses an explicitly family-centred approach, which includes two critical components: (1) addressing the health needs of the mother as well as the infant; and (2) recognizing that women's families should also be brought into care [27].

Integration of PMTCT services with HIV treatment and care not only facilitates women's access to care for her own HIV disease, but also improves the quality of PMTCT care by offering complex regimens [27] and by enriching the support context around the HIV-positive mother, who serves as the index case for this family-centred model.

A comprehensive package of services is offered to all family members and includes medical care for HIV-positive adults and children, early infant diagnosis, patient education and counselling, reproductive health and family planning services, psychosocial support, adherence and retention promotion, and nutrition education and support, as well as community outreach. These services are supported by a multidisciplinary team that includes nurses, physicians, counsellors, social workers, pharmacists and community health workers [27].

Access to related services is encouraged, including identification of and treatment for tuberculosis, nutritional support, family planning, and malaria prevention programmes. In addition to the broader scope of services, by offering treatment to all family members, long-term continuity of care, as well as treatment adherence, are promoted. For example, follow up of HIV-exposed infants is supported through programmes that ensure the availability of early infant diagnosis and treatment for children found to be HIV infected [27] (detailed outcomes of this study are provided in the results section and the summary of the programme model in Table 3).

#### 2. CDC-Uganda, Global AIDS Program

Using a home-based testing approach in rural Uganda, Mermin *et al *[28,29] evaluated several interventions that could be used to form a "preventive care package". Extension of VCT to 6000 family members of HIV-positive individuals was coupled with provision of cotrimoxazole for those found to be HIV-positive, as well as the distribution of more basic health interventions like home-based water purification systems. This study presents strong evidence of the benefits of addressing the health status of all family members.

Although the intervention was not specific to PMTCT, such approaches are readily applicable to family-centred PMTCT and speak to the potential for family-based interventions to have strong uptake and a "cascade" of positive effects within the family system (see detailed outcomes of this study in the results section and programme model summary in Table 3).

### Involving partners and other family members

One component of the family-centred approach that has been often overlooked in many programmes is the involvement of fathers and other members of the family in the prevention of vertical transmission. Our review indicates that father involvement has been credited with improved access to and retention of services, as well as improved health outcomes.

In the Ivory Coast, Tijou Traore *et al *[16] followed a cohort of HIV-positive women and their infants over a two-year period during a PMTCT project (see Table 4, Tijou Traore *et al*, ref [16]). When men knew that their spouse was HIV-positive and involved in the PMTCT project, they played an active role in applying the advice received, particularly related to exclusive breastfeeding and early weaning [16].

In Uganda, recognizing that male partners tended not to accompany women to prenatal visits and were often unlikely to take time off work, The AIDS Service Organization made efforts to increase uptake of HIV testing by offering special sessions on Saturdays for men [54]. However, positive outcomes associated with this strategy were not systematically documented. This shortcoming is indicative of the broader need for the development of an empirical evidence base highlighting the efficacy of such adaptive approaches to intervention.

Another novel programme in Uganda utilized an optout framework in order to increase uptake of HIV testing among men involved in their partner's antenatal clinic visits and delivery (see Table 1, Homsy *et al*). They found high levels of uptake of HIV testing at ANC visits (97% for women and men) and during delivery (86% for women and 98% for men). In addition, they observed a 12% increase in detection of HIV infection [21].

A fourth study, in Kenya, found that women accompanied by their partner for HIV-VCT were three times more likely to return for antiretrovirals; couples post-test counselling was also associated with an eight-fold increase in postpartum follow up, as well as greater antiretroviral utilization [22] (see Table 1, Farquhar *et al*, ref [22]).

Overall, it appears that engaging men has important benefits that support the goals of family-centred PMTCT. However, experiences across studies indicate that it remains challenging. Many successful programmes have relatively low rates of male engagement. For example, the study by Homsy *et al *[21], just described, demonstrated good participation from men, but only from those who were actively engaged in the care of their partners: among the 605 women who were tested in their study, only 180 of the men accompanied them at the time of delivery (30%). However, continued efforts to increase the involvement of men in family-based HIV testing, as well as counselling about infant feeding and child development, are likely to contribute to the effectiveness of family-centred PMTCT programmes.

### Going to scale via family-centred approaches to PMTCT

Family-centred PMTCT interventions have the potential to better engage families and to retain beneficiaries in care, thus creating a sound platform for the scale up of interventions. In fact, several national PMTCT programmes have utilized family-focused strategies to ensure successful scale up. For instance, Botswana is well known for its dramatic increase in PMTCT coverage: from 7% in 2000 to 83% by 2005. Family-centred components included integration of PMTCT with reproductive and child health services, psychosocial support for women, and ART for women's own clinical care [55].

Similar to the Botswana programme, the success of Thailand's PMTCT programme appears to be partially associated with its integration within a strong maternal and child health and public health programme, promoting close monitoring and follow-up care for women and HIV-exposed infants [56]. While both programmes demonstrate progressive models that integrate family-centred components of HIV care into existing systems, they could be further strengthened by extending services to additional family members.

### Ongoing challenges and advancing the field

Despite emerging examples of the power of family-centred approaches to PMTCT, and despite consensus among organizations like UNICEF and WHO that more holistic approaches are needed [2,57], there remains a prevailing focus on simplified medical interventions to reduce transmission. In reality, only a handful of studies investigate family-centred approaches to PMTCT. Given this state of affairs, and despite a compelling conceptual basis, the evidence base to move this agenda forward requires much more attention.

As a whole, current family-centred approaches remain largely underdeveloped and underdocumented. As seen in our review of available models, there are few formal published evaluations of family-centred PMTCT models and almost no comparative research in this area. Furthermore, although discussed as important, no programs reviewed here included direct attention to intimate partner violence, mental health issues or the integration of nutritional and early childhood development services into family-centred care. Therefore, trials pertaining to the efficacy of family-centred care versus "segmented delivery of only ART or PMTCT" are nearly non-existent [58].

## Conclusions

A paradigm shift is needed in PMTCT, which considers the needs of entire families, rather than placing a singular focus on preventing MTCT during pregnancy and delivery [15]. PMTCT represents an entry point for improving overall family health and functioning [27]. While family-centred models are relatively uncommon in the literature, those models that do exist show promising results.

These data speak to the prevailing perspective among stakeholders that a family-centred approach to HIV prevention and care is essential, compelling and far overdue, while also underscoring the continuing paucity of programmes and policies that actually work towards the realization of this ideal.

## Competing interests

The authors declare that they have no competing interests.

## Authors' contributions

TSB conceived the study, undertook research relevant to production of the manuscript, facilitated the screening process for study selection, and drafted or contributed to all parts of the manuscript. EJA helped to conceive the study, and undertook research relevant to production of the manuscript, in addition to editing and revising all parts. RM screened abstracts and studies for inclusion criteria, drafted the table of selected studies, and assisted in manuscript editing. MCSF helped to conceive the study and undertook research relevant to production of the manuscript, and drafted or contributed to all parts. All authors read and approved the final manuscript.
